# American Medical Association

**Published:** 1865-01

**Authors:** James T. Sevick

**Affiliations:** 1109 Arch St., Philadelphia


### AMERICAN MEDICAL ASSOCIATION.

The Committee of the American Medical Association, to which was referred, at its late annual meeting, the subject of "*Spotted Fever—so-called*," earnestly solicits from members of the Profession, in different parts of the country, information respecting the history, phenomena and treatment of this disease, as it has come under their own notice.

As it is desirable to learn the geographical distribution of this disease, intelligence respecting it, from physicians residing in distant States, and from Medical Officers of the Army, is particularly requested.

For the sake of uniformity, the following questions have been prepared. Any matters not included in, or suggested by them, will, with the answers themselves, be gratefully appreciated and acknowledged by the Committee.

Address the Chairman of the Committee.

DR. JAMES T. SEVICK,
1109 Arch St., Philadelphia.

1. When did "Spotted Fever—so-called," appear in your neighborhood, and how long did it prevail there?

2. What were the usual symptoms of the disease, and what unusual symptoms occurred in your practice?

3. Did it attack many individuals at the same time, and was it materially modified by the age, sex, or temperament of the patient?

4. What was the ordinary duration of the disease, and were relapses or second attacks common?

5. Are you in possession of any proof that the disease was communicated from one person to another?

6. What appeared to be the predisposing, and what the exciting causes of the disease?

7. What complications, and what sequelæ of the disease came under your notice?

8. What other diseases prevailed at or near the time that Spotted Fever did, and what epidemic diseases followed it?

9. What was your mode of treating the disease?

10. What was the proportion of deaths to the whole number of persons attacked, and what was the usual manner of fatal termination?

11. What were the *post mortem* appearances?

12. Were any microscopical observations made, and what were their results?

13. Has this disease prevailed in your neighborhood in former years?

